# Attention control in the peripartum period: a longitudinal study

**DOI:** 10.1007/s00737-024-01530-5

**Published:** 2024-11-11

**Authors:** Tamar Bakun Emesh, Nachshon Meiran, Dar Ran-Peled, Hamutal Ben-Zion, Avel Horwitz, Omer Finkelstein, Liat Tikotzky

**Affiliations:** Department of Psychology, Ben-Gurion University of the Negev, Beer Sheva, 84105 Israel

**Keywords:** Attention control, Peripartum period, Postpartum sleep, Postpartum cognition, Antisaccade task

## Abstract

**Purpose:**

Given research inconsistency, this study aimed to assess whether attention control changes from pregnancy to postpartum, focusing on the moderating role of maternal objective and subjective sleep. Our second objective was to evaluate attention control’s role in predicting psychological outcomes in peripartum women.

**Method:**

A cohort of 224 pregnant women completed the Antisaccade task, a measure of attention control, during the third trimester and again four months post-delivery. Objective and subjective sleep were measured using actigraphy and sleep diaries. Participants also completed questionnaires assessing depression, anxiety, emotion regulation, and maternal perceptions of the mother-infant relationship.

**Results:**

Attention control improved significantly from late pregnancy to postpartum (β = 0.91, *p* < .001). While objective sleep was not linked to attention control, poorer between-person subjective sleep was associated with better postpartum attention control (β = − 0.84, *p* < .001). Better within-person subjective sleep was associated with higher attention control during pregnancy (β = 0.87, *p* < .001), but a negative interaction with time (β = -1.5, *p* = .001) suggests a reverse trend postpartum. Attention control did not predict postpartum psychological outcomes.

**Conclusion:**

Cognitive recovery may occur by four months postpartum, although the observed improvement could reflect practice effect. The novel finding of a negative association between subjective sleep and postpartum attention control may indicate better adaptation to perceived poor sleep or heightened attunement to sleep fluctuations in women with higher attention control. Attention control did not predict psychological outcomes, suggesting other factors may be more critical for maternal coping postpartum.

## Article highlights

Contrary to expectations, attention control increased from late pregnancy to early postpartum, possibly indicating cognitive recovery or a practice effect.

Poorer subjective sleep was linked to better postpartum attention control, while objective sleep showed no significant association.

Antepartum attention control did not significantly predict postpartum sleep quality, depressive and anxiety symptoms, emotion regulation, or maternal perceptions of the mother-infant relationship.

While attention control is linked to parental functioning in other contexts, its predictive value during early motherhood appears limited, suggesting unique adaptive processes in this period.

The postpartum period is characterized by hormonal, biological, psychological, and social transformations (Lisofsky et al. 2019; Martínez-García et al. 2021; Skalkidou et al. 2012). Postpartum women face profound challenges, including changes in their social and personal lives, intensive infant caregiving demands, and a significant decline in sleep efficiency and continuity (Barba-Müller et al. 2019; Hipp et al. 2012; Insana et al. 2013; Yang et al. 2013).

Attention control (AC), which is also described by similar constructs such as inhibition, cognitive control, and executive control, may help cope with these challenges. AC enables individuals to selectively focus and actively maintain task-relevant information, guiding thought and action in the presence of internally or externally distracting information (Unsworth et al. 2021). Despite ongoing debates (Miyake and Friedman 2012; Rey-Mermet et al. 2018, 2019; Von Bastian et al. 2020), a recent mega-analysis (Unsworth et al. 2021) suggests AC as a coherent construct akin to the Common Executive Function described by Miyake and Friedman (2012; see Supplementary Materials), and is linked to critical cognitive abilities, mainly working memory capacity.

In their review, Crandall et al. (2015) found that mothers with better AC are more likely to engage in positive parenting behaviors and less likely to engage in harsh parenting behaviors. They suggest a conceptual model in which maternal AC directly impacts parenting, with evidence primarily derived from studies involving children aged toddler and above (Nordenswan et al. 2021; Rutherford et al. 2018; Yatziv et al. 2018). This has recently been suggested regarding adaptation in the postpartum period (Orchard et al. 2023).

However, it remains unclear whether AC itself is vulnerable to the drastic changes that occur after giving birth. Based on neuroimaging and cognitive research in rodents and humans, Orchard et al. (2023) proposed a theoretical framework suggesting a slight cognitive decline during pregnancy, primarily in the third trimester, followed by cognitive recovery in the late postpartum period. Although mixed evidence exists in the literature regarding when this recovery occurs (Kim et al. 2010; Lemaire et al. 2006; Pawluski et al. 2006), Orchard et al. (2023) suggested the weaning phase as the temporal mark of change. Accordingly, AC in the early postpartum is expected to be as compromised as in the third trimester of pregnancy. Considering that fatigue and sleep deprivation can affect individuals’ AC (García et al. 2021; Holanda Júnior and Almondes 2016) and that postpartum sleep is less efficient, more fragmented, and shorter compared to sleep during pregnancy (Hunter et al. 2009), one might expect postpartum AC to be degraded compared to antepartum. This hypothesis is supported by studies reporting declines in AC during the early postpartum period (Farrar et al. 2014; Glynn 2010; Insana et al. 2013).

Conversely, AC and other cognitive functions may not be affected during postpartum compared to during pregnancy or in control groups (Bannbers et al. 2013; Logan et al. 2014; Orchard et al. 2022; Rutherford and Anderson 2020). These findings align with AC’s consistency across the lifespan, underscored by its high heritability and stability of individual differences (Friedman et al. 2008, 2016; Tucker-Drob and Briley 2014). Some studies have also highlighted AC stability during situational fluctuations such as sleep deprivation (Killgore 2010) or hormonal changes (Dirk et al. 2020). These findings relate to the thesis of cultural expectations (Crawley et al. 2008; McCormack et al. 2023), suggesting that while objective peripartum cognitive decline is absent, peripartum women may erroneously perceive themselves as “less sharp” due to cultural stereotypes (Orchard et al. 2022; Rutherford and Anderson 2020).

Several methodological issues also contribute to the findings’ inconsistency. First, studies often fail to distinguish AC and other executive domains from general cognitive abilities like intelligence and memory (Henry and Sherwin 2012; Logan et al. 2014). An additional concern is the inconsistent operationalization of AC and its frequently used operationalization relying on tasks with low retest reliability, such as the Stroop, Flanker, and Tower of London tasks (Enkavi et al. 2019). Additionally, the operationalization of the postpartum period varies widely, from hours post-delivery to one year. According to Orchard et al.’s (2023) model, the mixed findings could be due to cognitive decline during pregnancy, with recovery during the late postpartum period. Lastly, small sample sizes limit the conclusions in some studies (Bannbers et al. 2013; Logan et al. 2014; Rutherford et al. 2018).

The main objective of the current study was to test two opposing hypotheses. The first hypothesis posits that postpartum women would show a decrease in their AC compared to during pregnancy and that this decrease would be related to sleep quality. The opposing hypothesis suggests the stability of objective AC, predicting no change over the peripartum period. The second objective was to explore the role of AC in predicting various psychological and behavioral outcomes in the postpartum period. We hypothesized that higher AC would predict better maternal objective and subjective sleep quality, maternal perceptions of the mother-infant relationships, emotion self-regulation, and lower depressive and anxiety symptoms.

## Method

### Participants and procedure

Data for this secondary analysis were extracted from a cohort study on sleep patterns of parents and infants during pregnancy and at 4, 8, and 12 months post-delivery (see Ran-Peled et al. 2022). Given our focus on the transition from pregnancy to early postpartum, we analyzed results from the third trimester of pregnancy and four months post-delivery.

Of 381 pregnant women recruited for the study, 224 completed the AC task during pregnancy (time 1). Table 1 shows demographics. Participants were visited at their homes by a research assistant, who administered the AC task and provided instructions on the study’s procedures, including actigraphy use, diary completion, and questionnaire filling. The study received approval from the Helsinki Committee of Soroka Medical Center, and all participants provided informed consent during the pregnancy assessment.

**Table 1:** Descriptive statistics of background covariates

	Age	Marital status	Maternal education	Household income
Mean (sd)	32.39 (5.42)			
Range	22–44			
%		Married 71.56 Solo-mother 28.44	High-school diploma 9.33 Bachelor’s degree 48.44 Master’s degree 40.00 Ph.D. degree 2.22	High below average 8.89 Below average 19.11 Average 21.78 Above average 42.67 High above average 7.11

Note. Solo-mother refers to single mothers by choice

### Measures

#### Attention control

**Fig. 1:**
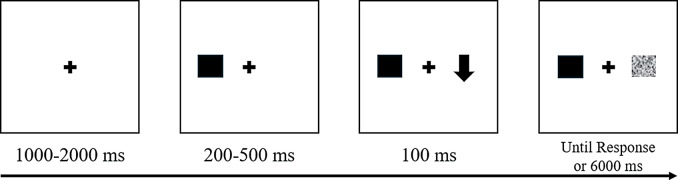
*The Antisaccade Task. Note* This task comprises 96 trials, following 24 practice trials. Each trial commenced with a central fixation mark (+) that appeared for a variable duration (1,000 m and 2,000 m in 500 m intervals). Subsequently, a cue was presented on one side of the screen (e.g., left) for 200 to 500 m in 100 m intervals, followed by the presentation of a target stimulus on the opposite side (e.g., right) for 100 m before being masked by gray cross-hatching that disappeared after response or after 6 s. The visual cue was a white square (64 × 64 pixels), and the target stimulus was a small white arrow (64 × 64 pixels). For further information regarding the task’s procedure and properties, see Supplementary Materials

AC was measured via the Antisaccade task, which requires participants to inhibit their automatic response towards a sudden-onset target and instead generate a voluntary response in the opposite direction (Fig. 1). Task retest intraclass correlation (ICC) ranges from 0.62 to 0.92 (Bakun Emesh et al. 2021; Ettinger et al. 2003; Meyhöfer et al. 2016; Wöstmann et al. 2013), indicating good-to-excellent retest reliability. In this study, the ICC(2,1) was 0.73.

#### Sleep assessment

Sleep was assessed both objectively and subjectively over seven nights. Objective assessment utilized actigraphy, a continuous recording of body movement, providing a reliable and valid method for assessing adult sleep-wake patterns (Ancoli-Israel et al. 2003). Here, we included four actigraphic sleep measures: sleep minutes (time from sleep onset to morning awaking, excluding periods of wakefulness during the night); sleep percent (percentage of sleep minutes out of the total sleep period); night waking (number of night-waking episodes lasting five minutes or longer); and longest sleep period (longest continuous sleep period without waking). Participants additionally filled out seven-night digital sleep diaries (matching the actigraphy measurement) to capture subjective evaluations of sleep-wake patterns. The diary-based measures included sleep minutes, sleep percent, number of wakings, and a total quality rating (scale: 1 = poor to 10 = excellent). Both objective and subjective measures were computed in terms of means and standard deviations, resulting in 8 measures per assessment (see further in Ran-Peled et al. 2022).

#### Maternal psychological outcomes

##### Depressive symptoms

The Edinburgh Postnatal Depression Scale (EPDS; Cox et al. 1987) consists of 10 short statements assessing depressive symptoms over the past week, each rated on a 4-point scale (0–3). The total score ranges from 0 to 30, with higher scores indicating greater severity.

##### Anxiety

The 20 state items from the State-Trait Anxiety Inventory (STAI; (Spielberger et al. 1971) were used, rating the frequency of feeling anxious on a 4-point scale (1–4), with total scores of 20–80 and higher indicating greater anxiety.

##### Emotion regulation

The Emotion Regulation Questionnaire (ERQ; Gross and John 2003) measures individuals’ tendencies to reappraise and suppress emotions, with ten items rated on a 7-point scale, each attributed to cognitive reassessment or emotional suppression.

##### Maternal perceptions of mother-infant relationships

Maternal Postnatal Attachment Questionnaire (MPAQ; Condon and Corkindale 1998) assesses maternal feelings toward the infant and the mother-infant relationship across four dimensions. With items rated on a 5-point scale, total scores range from 19 to 95, with higher scores indicating more positive perceptions.

In this study, Cronbach’s Alpha was 0.81/0.81 (Times 1 and 2) for EPDS, 0.90/0.91 (STAI), 0.73/0.73 (ERQ), and 0.64 (MPAQ).

### Statistical analysis

#### Antisaccade

Data were initially cleaned by removing responses quicker than 150 ms and slower than 3 SDs above the participant’s average, as well as practice trials. Before calculating reaction time (RT), error and post-error trials were also removed. Then, a modified Balanced Integration Score (BIS; Liesefeld et al. 2015) was computed as follows:

$$\:{BIS}_{i}=\:-{Z}_{{PE}_{i}}-{Z}_{\stackrel{-}{{RT}_{i}}}$$

Where $\:{Z}_{{PE}_{i}}$ is participant *i*’s standardized proportion of errors (PE), and $\:{Z}_{\stackrel{-}{{RT}_{i}}}$ is participant *i*’s standardized correct mean RT. Both $\:{Z}_{{PE}_{i}}$ and $\:{Z}_{\stackrel{-}{{RT}_{i}}}$ were computed using means and SDs from a large national-representative sample (*n* = 544, 278 males, mean age = 17.6 years, SD-age = 1, range 17–28).

In Bakun Emesh et al. (2021), BIS was more reliable than raw RT/error rate and other integration scores. BIS also allows one to include participants who lack mean RT in the final sample due to the high error rate, based on accuracy alone, as $\:-{Z}_{{PE}_{i}}$.

#### Principal components analysis (PCA)

**Fig. 2:**
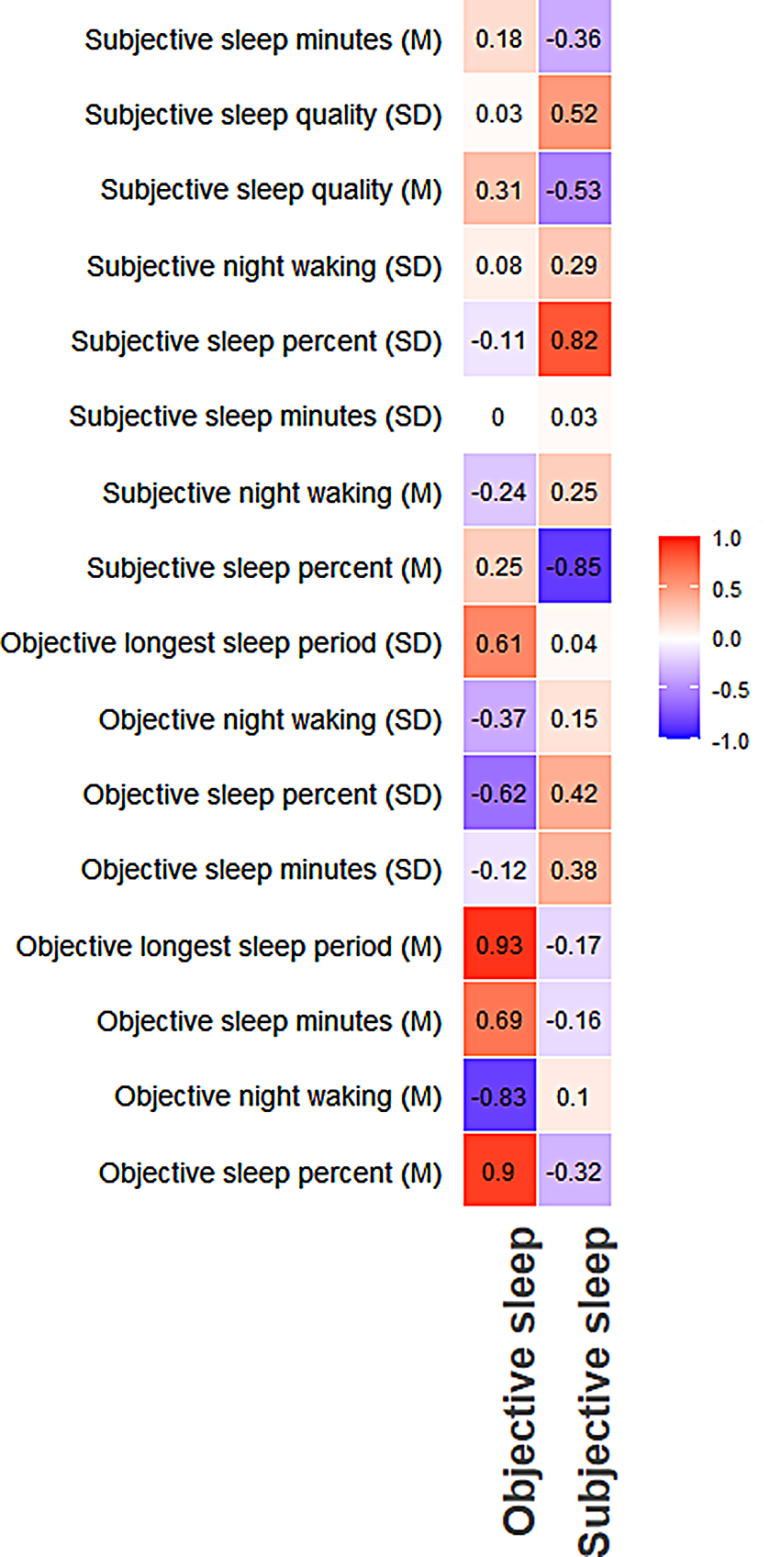
Components’ Loading on Actigraphy (Objective) and Diary (Subjective) Sleep Measurements

The study included 16 sleep variables. To reduce data dimensionality without significant information loss, we conducted PCA, a widely used technique for this purpose (Zhang and Castelló 2017). We conducted PCA with oblique (PROMAX) rotation, using sleep data from Time 1 (N = 274), regardless of participants’ completion of the Antisaccade task, to endure stability. PCA was conducted using “psych” package (Revelle and Revelle 2015), in R software (R Core Team 2013). The number of components was determined to be two based on the scree-plot. Figure 2 presents a heatmap of all variables’ loadings on the two components. Note that PCA is data-driven, yet when looking at the variables’ loading, one may notice a pattern in which subjective and objective sleep have distinct variances. Component 1 (explaining 24% of the variance), labeled ‘objective sleep,’ loaded mainly by actigraphy measures, with higher scores indicating better sleep. Component 2 (16%), ‘subjective sleep,’ loaded mainly by self-report measures, with higher scores indicating poorer sleep, but inverted for consistency with Component 1. The two-component scores were then calculated for each participant at each time point.

#### Multi-level modelling (MLM) for longitudinal data

We employed an MLM to investigate changes in AC over time and their relationship to sleep. First, we constructed an unconditional model that predicted AC as a function of time, treating time as a within-person variable. Next, we estimated the effects of each sleep component in two ways: as a within-person variable and a between-person variable. The sleep variable was centered using person-mean centering. The within-person effect examined how deviations from a participant’s average sleep (both objective and subjective) at a specific time point related to AC at that time. Additionally, we explored whether the relationship between time and AC was moderated by these deviations in sleep. The between-person effect examined how a participant’s deviation from the sample’s average sleep (both objective and subjective) related to the participant’s AC at Time 2. We also examined whether the relationship between time and AC varied depending on the between-person differences in sleep. For this analysis, we employed data from participants who completed the Antisaccade both in Time1 and in Time2 (*N* = 135).

#### Hierarchical regression models

To assess whether AC predicts sleep and various postpartum psychological outcomes, we conducted a series of hierarchical regression models; in each, we predicted one outcome in three steps: Step1 included only background covariates (i.e., age, marital status, income, and education); in Step 2 we added the outcome variable as it was measured in Time1, to control for baseline levels of the outcome; finally, in Step 3, prenatal Antisaccade was added as a predictor (Note: For MPAQ prediction, only Steps 1 and 3 were conducted, as there was no MPAQ score at Time1). Since within-sample estimates are prone to overfitting, we assessed a linear regression model’s out-of-sample R^2^ and R^2^ change using 200 repetitions of 5-fold cross-validation, utilizing ‘caret’ package (Kuhn 2009).

## Results

### Change over time in AC

See Table 2 for correlations between the study’s variables and Table 3 for descriptive statistics. Table 4 shows the five MLMs. Participants performed better at Time 2 than at Time 1 (β = 0.91, *p* < .001 in the unconditional time model). Poorer postpartum between-person subjective sleep predicted better Antisaccade performance (β = − 0.84, *p* < .001), with no interaction effect between time and sleep. Additionally, the between-person analysis found no significant association for postpartum objective sleep. When sleep was treated as a within-person variable, better subjective sleep at Time 1 predicted better Antisaccade performance at Time 1 (β = 0.87, *p* < .001). A significant interaction between time and subjective sleep was also found (β = -1.5, *p* = .001), suggesting that the initial positive effect observed at Time 1 reversed at Time 2, where relatively poorer subjective sleep was associated with better Antisaccade performance. No significant association was found between within-person objective sleep and Antisaccade performance.

**Table 2:** Pearson/Spearman/bi-serial correlations between the study’s variables at pregnancy (Time 1) and 4 months Postpartum (Time 2)

	Age		Marital status		Maternal education		Household income	
	Time 1	Time 2	Time 1	Time 2	Time 1	Time 2	Time 1	Time 2
Antisaccade	− 0.27***	− 0.40***	− 0.18**	− 0.33***	0.04	− 0.03	0.07	0.05
OS	− 0.16	0.036	− 0.10	− 0.001	0.07	− 0.001	0.05	0.16*
SS	0.26***	− 0.39***	0.27***	− 0.37***	− 0.05	− 0.09	− 0.04	− 0.06
EPDS	− 0.07	0.04	− 0.07	0.01	.-0.06	− 0.04	− 0.01	− 0.01
STAI	0.10	0.05	0.05	− 0.05	− 0.03	0.002	− 0.02	0.08
ERQ (es)	0.10	− 0.06	0.11	− 0.01	0.02	− 0.08	− 0.05	− 0.10
ERQ (cr)	0.03	− 0.08	0.10	0.02	− 0.02	− 0.04	− 0.08	− 0.08
MPAQ	-	− 0.20**	-	− 0.16*	-	− 0.07	-	− 0.07

Note *p < .05. **p < .01. ***p < .001. OS = Objective Sleep; SS = Subjective Sleep; EPDS = Edinburgh Postnatal Depression Scale; STAI = State-Trait Anxiety Inventory; ERQ (cr) = Emotion Regulation Questionnaire, cognitive reappraisal; ERQ (es) = Emotion Regulation Questionnaire, emotion suppression; MPAQ = Maternal Postnatal Attachment Questionnaire. Note that marital status was coded as 1 = married, 2 = solo-mother (*single mothers by choice). Maternal education and household income were both coded as ordinal variables, with the lowest level coded as 1

**Table 3:** Descriptive statistics of the study’s variables

	*N*	Mean (sd)	Range	Kurtosis	Skewness
Antisaccade _time 1_	224	-1.005 (2.079)	-8.704-3.470	4.717	− 0.999
Antisaccade _time 2_	134	− 0.007 (1.819)	-7.003-3.023	-1.110	4.654
OS _time 2_	179	− 0.002 (0.604)	-2.275-1.219	3.299	− 0.417
SS _time 2_	166	0.009 (0.650)	-2.062-1.442	3.400	− 0.468
EPDS _time 2_	195	4.510 (3.620)	0–16	3.217	0.928
STAI _time 2_	193	34.830 (9.004)	20–64	3.405	0.854
ERQ (cr) _time 2_	190	27.576 (6.982)	6, 42	3.688	− 0.354
ERQ (es) _time 2_	190	10.241 (4.403)	4–25	2.943	0.620
MPAQ _time 2_	192	79.358 (6.517)	46–95	6.724	-1.104

Note OS = Objective Sleep; SS = Subjective Sleep; EPDS = Edinburgh Postnatal Depression Scale; STAI = State-Trait Anxiety Inventory; ERQ (cr) = Emotion Regulation Questionnaire, cognitive reappraisal; ERQ (es) = Emotion Regulation Questionnaire, emotion suppression; MPAQ = Maternal Postnatal Attachment Questionnaire

**Table 4:** MLM results Predicting Antisaccade performance

	Unconditional Time Model	Time*between-person OS Model	Time*between-person SS Model	Time*within-person OS Model	Time*within-person SS Model
Fixed effects:	*B (SE)*	*B (SE)*	*B (SE)*	*B (SE)*	*B (SE)*
Intercept	− 0.92*** (0.17)	− 0.86*** (0.17)	− 0.86*** (0.17)	**− 0.76 (0.16)**	− 0.83*** (0.16)
Time	0.91*** (0.12)	0.89*** (0.12)	0.99*** (0.12)	**0.84 (0.12)**	0.98*** (0.12)
OS		− 0.01 (0.29)		0.60 (0.33)	
Time*OS		− 0.16 (0.20)		− 0.75 (62)	
SS			− 0.84*** (0.24)		0.87*** (0.25)
Time*SS			0.27 (0.18)		-1.5** (0.46)
Variance components:	Var (*SE*)	Var (*SE*)	Var (*SE*)	Var (*SE*)	Var (*SE*)
Residual	0.95 (0.98)	0.86 (0.93)	0.86 (0.93)	0.84 (0.92)	0.84 (0.92)
Intercept	2.93 (1.71)	2.54 (1.59)	2.40 (1.55)	2.27 (1.51)	2.13 (1.46)
BIC	1044.67	927.14	899.89	838.59	848.40
N	135	122	119	112	114

Note *p < .05. **p < .01. ***p < .001. Note. OS = Objective Sleep; SS = Subjective Sleep

To further explore the robust main effect of time on AC, we analyzed the trend over the entire study period, including additional time points at 8 and 12 months postpartum. Despite significant dropout (*N* = 226, 136, 91, and 50 across the four time points), a post-hoc analysis revealed a significant positive trend in Antisaccade performance from late pregnancy through the first year postpartum (see Supplementary Materials). Importantly, this effect may be attributed to a practice effect (Pieters et al. 2021). To account for this, we compared the change in Antisaccade performance in this study with the change in another sample (Gordon et al. 2018). In this sample, 135 participants (85 males, mean age = 22.16 years, SD age = 2.6, range 19–28) completed the Antisaccade task in two sessions, separated by a time interval of 5 weeks. We found a main effect for time (*F(1)* = 27.601, *p* < .001) and for study (*F(1)* = 110.859, *p* < .001), without an interaction between time and study (*F(1)* = 0.367, *p* = .545). This finding implies that the increase in Antisaccade from Time1 to Time2 could be attributed to practice (see Supplementary Materials).

### Antisaccade as a predictor of postpartum outcomes

Table 5 shows the results of seven hierarchical regression models for each psychological outcome. When added as Step 2 variables, the baseline scoring of the STAI (β = 0.78, *p* < .001, 95%CI[0.13, 0.77]) and the ERQ scales (β_ERQcr_ = 0.58, *p* < .001, 95%CI[0.01, 0.58]; β_ERQes_ = 0.68, *p* < .001, 95%CI[0.19, 0.61]) significantly contributed beyond background covariates, indicating that levels during pregnancy are associated with postpartum levels. In contrast, objective and subjective sleep measures and EPDS scores at Time 2 did not significantly improve the prediction of outcome variables. Importantly, Antisaccade did not predict any of the time 2 outcomes.

**Table 5:** Hierarchical regression analyses for Predicting Postpartum Psychological outcomes

	OS	SS	EPDS	STAI	ERQcr	ERQes	MPAQ
Step1							
Age	0.008	− 0.027	0.008	0.540*	− 0.412*	− 0.083	− 0.175
Marital status	− 0.003	− 0.454	− 0.698	-6.269*	4.374	0.450	-1.012
Income	0.101*	− 0.057	− 0.035	0.163	− 0.048	− 0.311	− 0.489
Education	− 0.061	− 0.053	− 0.245	− 0.378	− 0.225	− 0.245	− 0.278
R^2^ (mean)	0.032	0.164	0.040	0.028	0.026	0.017	0.037
Step 2							
Outcome _time1_	0.141**	− 0.009	0.349***	0.777***	0.582***	0.681***	
R^2^ (mean)	0.049	0.126	0.109	0.541	0.301	0.412	
ΔR^2^ (95%CI)	0.017 (-0.124, 0.170)	− 0.038 (-0.361, 0.274)	0.069 (-0.110, 0.279)	**0.513** **(0.125**,** 0.768)**	**0.275** **(0.010**,**0.575)**	**0.395** **(0.187**,** 0.605)**	
Step 3							
Antisaccade	− 0.025	0.084	0.349	− 0.105	0.254	− 0.149	− 0.133
R^2^ (mean)	0.068	0.202	0.100	0.538	0.300	0.409	0.030
ΔR^2^ (95%CI)	− 0.001 (-0.184, 0.196)	0.076 (-0.244, 0.396)	− 0.009 (-0.235,0.211)	− 0.003 (-0.464, 0.472)	− 0.001 (-0.378, 0.377)	− 0.002 (-0.378, 0.377)	− 0.005 (-0.130, 0.104)

Note *p < .05. **p < .01. ***p < .001. OS = Objective Sleep; SS = Subjective Sleep; EPDS = Edinburgh Postnatal Depression Scale; STAI = State-Trait Anxiety Inventory; ERQcr = Emotion Regulation Questionnaire, cognitive reappraisal; ERQes = Emotion Regulation Questionnaire, emotion suppression; MPAQ = Maternal Postnatal Attachment Questionnaire. Note that marital status was coded as 1 = married, 2 = solo-mother. Maternal education and household income were both coded as ordinal variables, with the lowest level coded as 1

## Discussion

AC may help cope with postpartum challenges, but biological changes and sleep disturbances may affect it. The primary finding in this study is a significant increase in AC from late pregnancy to 4 months postpartum, possibly reflecting a cognitive renormalization in this phase (Orchard et al. 2023), in line with previous research showing cognitive decline up to 3 months postpartum (Farrar et al. 2014; Glynn 2010; Henry and Sherwin 2012; Insana et al. 2013). The observed increase in AC over 12 months postpartum strengthens this conclusion but should be interpreted cautiously due to the significant number of dropouts and the fact that the difference seen may reflect practice effect. The fact that participants were tested at home enhances the ecological validity of this conclusion (McCormack et al. 2023).

Our findings did not reveal significant links between objective sleep and AC, contrasting with previous research (Chary et al. 2020). Interestingly, we observed associations between AC and subjective sleep: Women who perceived their sleep during pregnancy as better showed higher AC during that time, but this trend reversed during the postpartum period, resembling the across-time picture where women who perceived their sleep as poorer exhibited higher AC. These findings suggest a surprisingly negative association between subjective sleep and AC. It may be that subjective feelings of poor sleep and lack of resources might lead to the recruitment of AC. This aligns with research suggesting that the maternal brain adapts to motherhood demands (McCormack et al. 2023). Alternatively, women with higher AC may be more attuned to subtle changes in their sleep patterns, independent of the peripartum context. This explanation is in line with previous research linking AC to self-reflection (İlkmen and Soncu Büyükişcan 2024), specifically in mothers (Yatziv et al. 2018). Clearly, additional research is needed to replicate and account for these findings.

We found no significant prospective connections between AC during pregnancy and various postpartum psychological outcomes, including sleep and anxiety symptoms, perceived mother-infant relationship quality, and emotion regulation. These findings may indicate that maternal adaptation during early motherhood presents a unique scenario. Perhaps social cognition, rather than AC, plays a more significant role in maternal experiences (Anderson and Rutherford 2012; Barba-Müller et al. 2019), possibly seen in the gray matter volume reduction during pregnancy, especially in areas related to Theory of Mind (ToM) and responses to infant stimuli, suggesting adaptive transitioning into motherhood (Hoekzema et al. 2017). Importantly, these changes do not seem to involve brain regions associated with AC (Cieslik et al. 2015).

Study limitations should be considered. First, the current analysis lacks baseline measurement before pregnancy, which restricts our ability to accurately conclude if there were any real changes from pre-pregnancy to postpartum. Similarly, the absence of a nulliparous control group limits our ability to robustly assess the suggestion of a practice effect. Second, a more comprehensive assessment of AC and other cognitive functions would have strengthened our conclusions. Third, psychological outcomes were measured using self-reports with inherent limitations. Fourth, our sample does not fully represent the general population in terms of demographic and psychological characteristics.

## Conclusion

Our results indicate that AC improves in the postpartum period compared to pregnancy, suggesting that cognitive renormalization may begin by four months postpartum. The observed trend may be influenced by practice effects and subjective sleep perceptions. Furthermore, our findings suggest AC does not significantly predict sleep and various psychological aspects during the postpartum period.

## Data Availability

Data will be made available on request.

## Declarations

### Pre-registration

This study was pre-registered before data analysis (registration: https://doi.org/10.17605/OSF.IO/YRCPX).
